# Post-pandemic pain services: a new world

**DOI:** 10.1177/2049463720942663

**Published:** 2020-08

**Authors:** Paul Cameron

The first half of 2020 has seen a significant change in the way we work, live and play and, for many, has forced us to evaluate the most important things in life. Our priorities and goals for the future may have changed, and the way we perceive our work/life balance may well have altered substantially. Likewise, the National Health Service (NHS), regardless of whether in a devolved nation or not, has seen a fundamental shift in its capacity and capability and an increase in the demand on its resources in a manner unprecedented and at a scale never seen before, altering the way both clinicians and patients view the NHS. Pandemic viruses know no boundaries and do not respect devolution or national pride, and we have seen the different approaches taken across the United Kingdom and the effect this has had on the NHS. However, regardless of location it is notable that pain services, covering both acute and chronic pain populations have all suffered the same fate. Doctors re-assigned to concentrate on other clinical areas, and physiotherapists, occupational therapists, psychologists, nurses and pharmacists, and a multitude of others, all moved to support intensive care and high dependency areas, wards and the community in order to play their part in the pandemic response.

The significant reduction in resources to pain service provision did not see the disappearance of the need for such services, and it is a concern for many clinicians as to the impact the pandemic response will have on patients now and in the future. Indeed, it was a testament to the professionalism and caring nature of staff remaining in such clinics who developed innovative ways to provide some degree of support to patients. Digital technology featured highly as a new approach, with telephone and video consultations utilised for triage, assessment, and reviews; roll-out and updates of IT hardware and software across the United Kingdom; and the speedy creation and compilation of online resources to be offered to patients. However, little is known of the efficacy or sustainability of such approaches or of the impact it will have on patients unable, or unwilling, to access such technology.

As lockdown eases across the United Kingdom, pain services will gradually increase their resources and ability to assess and treat patients.^1^ However, it is unlikely that these will be uniform across regions, and ongoing social distancing will have a direct effect on the capacity clinics have available to them to see patients and, indeed, accommodate their staff. The expectation that clinics will take the opportunity to review their approaches will no doubt be a significant factor in ongoing commissioning and service reviews and it is clear that the current use of digital technologies will be here to stay, despite their initial emergency instatement.^2^

This issue of the *British Journal of Pain* contains a number of interesting articles relevant to the clinical treatment of pain. As pain services slowly rise out of the ashes into a new, post-COVD-19 NHS, taking on a new persona reflective of new circumstances, those articles exploring the predictors of first-appointment attendance at a pain service, smartphone technologies, and digital pain programmes are likely to hold particular relevance. Going forward, it will be important that any new initiatives, and innovative technology, utilised to assist in treating patients digitally or otherwise remotely are evaluated carefully addressing both quantitative outcomes related to easing of pain and the qualitative experience of patients. Journals such as this, with a focus on pain, will take on an important role in the timely dissemination of conclusions from these studies to allow clinicians to quickly apply and adapt such findings to their daily clinics.

However, in this brave new world of innovation in pain services, questions remain as to how the ethics of new approaches will be taken into account and how we will ensure that patients continue to receive equitable and appropriate care, in a manner that they will best engage with. It would be a retrograde step to apply a utilitarian approach^3^ to treatments simply to meet demand in areas with reduced capacity and a markedly increased waiting list. Indeed, to do so would likely increase difficulties faced by both patients and clinicians as approaches applied inappropriately would lead to an increase in chronicity and patients re-entering specialised services, together with subsequent increases in their use of primary care services.

Accordingly, I encourage anyone conducting research to consider how it will apply in real time to patients and clinicians on the ground navigating their way through this new, unchartered and often digital, territory. By examining the utility of new interventions closely, and considering their long-term impact on our patient population, we can ensure that while we embrace new ways of thinking and technologies the people who need our help the most remain at the centre of our efforts.


**Paul Cameron** Division of Population Health Science and
Genomics, University of Dundee, UK; School of Medicine, Cardiff University, UK; NHS
Fife Pain Service, Queen Margaret Hospital, Dunfermline, Fife, UK

